# Spectral Similarity Assessment Based on a Spectrum Reflectance-Absorption Index and Simplified Curve Patterns for Hyperspectral Remote Sensing

**DOI:** 10.3390/s16020152

**Published:** 2016-01-26

**Authors:** Dan Ma, Jun Liu, Junyi Huang, Huali Li, Ping Liu, Huijuan Chen, Jing Qian

**Affiliations:** 1 College of Resource and Environmental Science, Fujian Agriculture and Forestry University, Fuzhou 350002, China; madam_yurou@163.com; 2 Shenzhen Institutes of Advanced Technology, Chinese Academy of Sciences, Shenzhen 518055, China; liuping@siat.ac.cn (P.L.); hj.chen@siat.ac.cn (H.C.); jing.qian@siat.ac.cn (J.Q.); 3 Department of Geography, Hong Kong Baptist University, Hong Kong, China; jrhuang@life.hkbu.edu.hk; 4 College of Electrical and Information Engineering, Hunan University, Hunan 410082, China; lihuali@hnu.edu.cn

**Keywords:** similarity assessment, spectrum absorption-reflection idex, simplified curve pattern, Douglas-Peucker algorithm, hyperspectral remote sensing

## Abstract

Hyperspectral images possess properties such as rich spectral information, narrow bandwidth, and large numbers of bands. Finding effective methods to retrieve land features from an image by using similarity assessment indices with specific spectral characteristics is an important research question. This paper reports a novel hyperspectral image similarity assessment index based on spectral curve patterns and a reflection-absorption index. First, some spectral reflection-absorption features are extracted to restrict the subsequent curve simplification. Then, the improved Douglas-Peucker algorithm is employed to simplify all spectral curves without setting the thresholds. Finally, the simplified curves with the feature points are matched, and the similarities among the spectral curves are calculated using the matched points. The Airborne Visible Infrared Imaging Spectrometer (AVIRIS) and Reflective Optics System Imaging Spectrometer (ROSIS) hyperspectral image datasets are then selected to test the effect of the proposed index. The practical experiments indicate that the proposed index can achieve higher precision and fewer points than the traditional spectral information divergence and spectral angle match.

## 1. Introduction

A hyperspectral remote sensing image has the combined characteristics of an image and spectrum; in addition, undetectable substances can be easily detected in multi-spectral remote sensing images [1]. In recent years, hyperspectral remote sensing technologies have developed rapidly and are being widely applied in many industries. Remote sensing image classification has evolved from rough recognition using multi-spectral images to undertake spectrum-analysis-based fine identification using hyperspectral images [2,3]. However, due to the increased cost of hyperspectral remote sensing image data volume, high correlation among the bands and training samples, the traditional hyperspectral remote sensing image classification and recognition technologies cannot satisfy the requirements of hyperspectral remote sensing applications [4]. 

To address issues in hyperspectral remote sensing image feature identification and classification, scholars have proposed various methods, which can be roughly divided into two categories: ones based on feature space and those based on spectral space. For the first category, according to the Hughes phenomenon, the number of training samples can sharply increase with the increase of band number. The common solution is to reduce the dimensionality of the hyperspectral image by using the methods of principal component analysis [5,6], independent component analysis [7], information entropy [8], genetic algorithm, projected track [9], wavelet analysis [10], minimum noise fraction transformation [11], neural networks [12], particle swarm optimization, manifold learning algorithm, and so on. The Hughes phenomenon can be avoided once the dimensionality of the hyperspectral remote sensing image is controlled. The hyperspectral remote sensing image classification can be completed using conventional remote sensing image classification methods, such as the maximum likelihood method [13], Bayesian classifier method [14], decision tree, artificial neural networks [15], and so on, Another interesting classification approach is based on kernel space, and includes support vector machines (SVM) and kernel fisher discriminates (KFD), to name a few. Such methods have been widely used because they are not limited by the Hughes phenomenon [16].

The other category is based on spectral space, and includes Euclidean distance (ED), spectral absorption index (SAI), spectral angle mapper (SAM) [17,18], spectral correlation mapper (SCM), mutual information [19], spectral gradient angle [20] spectral information divergence (SID) [21], set methodology, and so on. Based on the fine spectral information of hyperspectral remote sensing images, such methods do not require complex analysis and dimension reduction. 

As the essence of remote sensing image classification is to divide objects into several different categories by extracting the characteristics of different objects and using a certain similarity measurement combining some classification criteria, the key aspect of hyperspectral remote sensing image classification is the assessment of spectral similarities among different objects. Some scholars have carried out impact studies on the similarity assessment of the classification results by combining spectral amplitude and shape. 

Du *et al*. [22] proposed a classification method combining the spectral information dispersion and spectral cosine angular, while Kumar *et al*. [23] proposed a method using the combination of the correlation coefficient (CC) and spectral information dispersion. Kong *et al*. [24] presented a spectral similarity metric that combines various spectral features using geometric distance, the CC, and relative entropy. Meanwhile, Du *et al*. [25] extracted the characteristics of spectral absorption/reflection, central moment, fractal dimension and information entropy for image retrieval, and concluded that these are actually inappropriate parameters for image retrieval. Spectral curve feature extraction has also been applied to various fields, such as crop identification and chemistry analysis [26,27]. SAM has been used extensively for distinguishing different objects because it is capable of repressing the influence of shading to enhance the target reflectance [28]. However, this method can only distinguish between negative and positive correlations because only the absolute value is considered [29]. In comparison, SID models the spectrum as a probability distribution and describes the spectral features by using statistical moments; it also considers the spectral variability as a random uncertainty [21]. Based on these advantages, some studies have combined SAM and SID to increase the discriminatory capacity for effective image retrieval [30]. These studies have proven that the classification accuracy of using a combination of two or more similarity measures is higher than that using a single similarity measure. However, the comparisons and evaluations of similarity measures have been carried out using only spectral data or a few samples of land cover types. Furthermore, the comparison methods do not use uniform standards and, to date, there have been few systematic evaluations of similarity measure methods.

All the bands of reference and measured spectra are involved in the computation when using various similarity metrics. In this case, the enormous amount of required calculation can lead to low efficiency in similarity assessment because there may be hundreds of thousands of bands involved. Thus, certain simplified or filtration methods must be used to reduce the amount of computation. The Douglas-Peucker (DP) algorithm is an efficient and extensively used method to simplify a curve. However, the simplification effect depends on the threshold, which cannot be adaptively set. Therefore, in this paper, an improved DP (IDP) algorithm without threshold under spectral reflection-absorption index (SRAI)-restriction is proposed to meet this requirement. In addition, a similarity index is proposed to assess the similarities among different spectrum curves. Section 2 reviews two widely used similarity metrics, *i.e.*, SAM and SID. Section 3 presents the proposed SRAI-restrained IDP method. Section 4 illustrates the assessing experiments using the proposed method and traditional ones. Then, the effects of the parameters are evaluated. The final section draws the conclusions based on the obtained results.

## 2. Related Similarities of Spectral Vectors

A spectral vector (curve) is the representation of ground feature that is used in hyperspectral remote sensing. Ground feature identification can be achieved by measuring the similarities of the spectral vectors. Two typical similarity indices are shown below.

### 2.1. SID

SID is based on the information entropy; it can measure the similarities and separability of pixels. First, the information entropy of each point is calculated; then, they are compared to measure the similarities of the spectral vectors using the formula:

(1) SID(A,B)=D(A||B)+D(B||A)

where:

(2) $$D(A||B)=-\sum_{i=1}^{N}p_{i}\log(p_{i}/q_{i})$$

(3) $$D(B||A)=-\sum_{i=1}^{N}q_{i}\log(q_{i}/p_{i})$$

(4) $$p_{i}=A_{i}/\sum_{i=1}^{N}A_{i}$$

(5) $$q_{i}=B_{i}/\sum_{i=1}^{N}B_{i}$$

In the equations above, *N* refers to the number of bands, and $A=(A_{1},A_{2},\cdot \cdot \cdot ,A_{N})$ and $B=(B_{1},B_{2},\cdot \cdot \cdot ,B_{N})$ refer to the two spectral vectors, respectively. Here, the lower the SID value, the higher the similarity of both spectral vectors.

### 2.2. SAM

SAM, also known as vector-included angle cosine method, assess similarity by calculating the angle between two spectral vectors. The spectral angle refers to the angle of two spectral vectors with the same wavelength range, which is given by:

(6) $$\cos\alpha =\frac{A\cdot B}{\left|A||B\right|}=\sum_{i=1}^{N}A_{i}B_{i}/(\sqrt{\sum_{i=1}^{N}A_{i}A_{i}}\sqrt{\sum_{i=1}^{N}B_{i}B_{i}})$$

where *A* and *B* refer to the two spectral vectors, respectively, and *α* refers to the spectral angle. The cosine value of *α* is needed in the computation. Here, the higher the SAM value, the higher the similarity of the two spectral vectors. 

## 3. Proposed Approach

### 3.1. SRAI

The spectral curves of various ground features have different properties in terms of absorption wave peak and valley, location, width, depth, and symmetry. Therefore, SRAI can be used to illustrate the spectral curve identification feature. 

**Figure 1:**
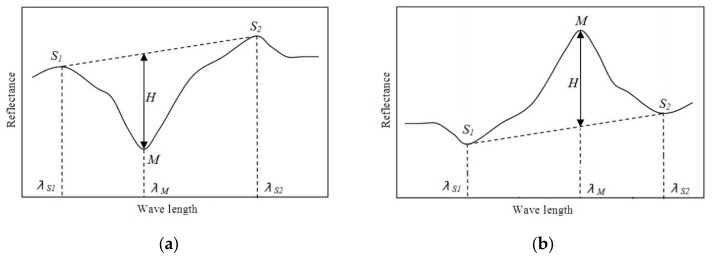
Illustrations of SRAI (**a**) absorption and (**b**) reflection.

Spectral absorption is characterized by the valley (M) and its corresponding peaks (*S*_1_ and *S*_2_), or the peak and its corresponding valleys valleys as shown in Figure 1. *H* represents the spectral absorption depth, and is the distance between the non-absorption baseline formed by valley (M) and its corresponding peaks. Let us suppose that$\rho_{S1}$, $\rho_{S2}$, and $\rho_{M}$ are the reflectances of *S*_1_, *S*_2_ and *M*, respectively, and $\lambda_{S1}$, $\lambda_{S2}$ and $\lambda_{M}$ are the wavelengths of *S*_1_, *S*_2_, and *M*, respectively. Thus, the width of absorption band, asymmetry parameter, and the reflectance rate of peak can be respectively obtained using the expressions:

(7) $$W=\lambda_{S2}-\lambda_{S1}$$

(8) $$d=\frac{\lambda_{S2}-\lambda_{M}}{W}$$

(9) $$\Delta \rho_{S}=\rho_{S2}-\rho_{S1}$$

Therefore, the formula of the non-absorption baseline is:

(10) $$W\cdot \rho -\Delta \rho_{S}\cdot \lambda =\rho_{S1}-\Delta \rho_{S}\cdot \lambda_{S1}$$

Equation (10) expresses the spectral contribution and spectral behavior of ground features without the spectral absorption characteristics. In this case, SRAI can be defined as the ratio of the spectral value of absorption and the corresponding baseline, as expressed by:

(11) $$SRAI=\frac{d\rho_{S1}+(1-d)\rho_{S2}}{\rho_{M}}$$

**Figure 2:**
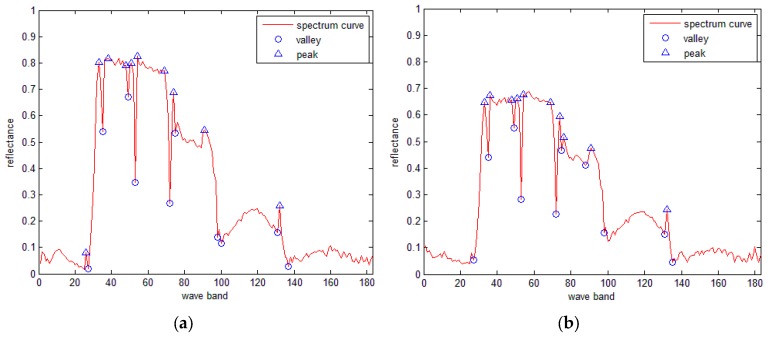
The SRAI features extracted from the spectral curves of a (**a**) tree and (**b**) wheat.

Figure 2 shows the spectral curves of tree and wheat from an AVIRIS hyperspectral image, from which 20 SRAI feature points (including 10 valley points and 10 peak points) are extracted.

### 3.2. Classical DP Algorithm

Hundreds of bands are involved in calculating similarities among hyperspectral images. One possible way to reduce the computation load is to simplify the spectral vector. The DP algorithm, vertical distance algorithm, and Li-Openshaw algorithm are among the most common methods used, of which the DP algorithm is the most popular. The basic principles of using the DP algorithm are listed below:

(1) Connect both ends of the curve with a straight line and calculate the distances (d) from all the points on curve to the line.
(2) Compare the maximum distance (*dmax*) and threshold *D*; if *dmax* < *D*, then eliminate all points on the curve; otherwise, retain the point with the maximum distance (*dmax*) and split the curve into two parts;
(3) Repeat steps 1 and 2 until *dmax* < *D* is true for all points on the curve, and all the retained points comprise the final simplified spectral curve.

**Figure 3:**
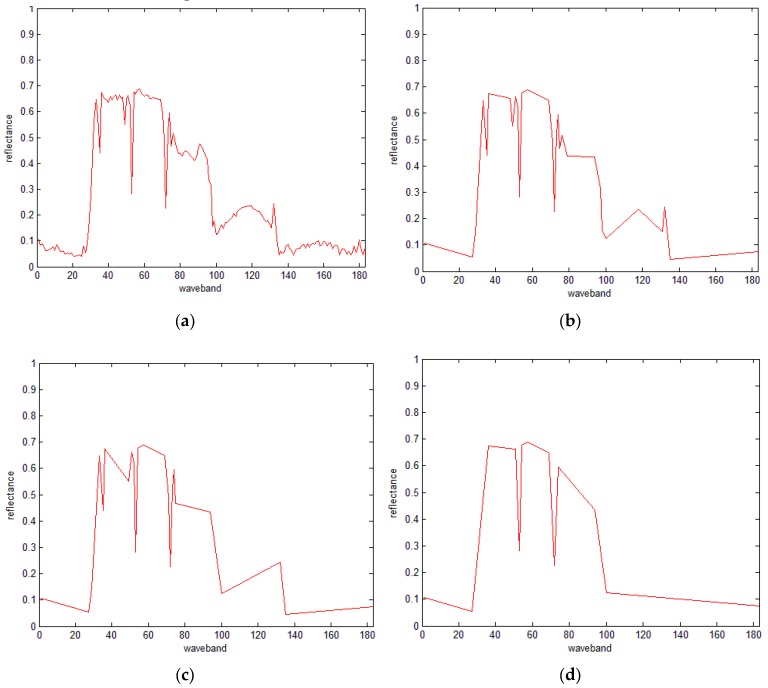
The simplified spectral curve using the DP algorithm with different thresholds. (**a**) The original spectral curve with 183 points, (**b**) *D* = 0.05, 30 retained points, (**c**) *D* = 0.1, 23 retained points, and (**d**) *D* = 0.2, 13 retained points.

Figure 3 illustrates the simplified spectral curve using the DP algorithm with different thresholds (*D*). As can be seen, as the threshold increases, more points are removed and the spectral curve becomes more simplified.

The DP algorithm is able to preserve the curve pattern feature; however, recursion is needed for processing, which further complicates the process. This process also has three main disadvantages, which are listed below:

(1) The threshold must be specified as it can significantly affect the performance of curve simplification. On the one hand, if the threshold becomes higher, fewer points are retained, which may destroy the pattern. On the other hand, if the threshold becomes lower, more points are retained; therefore, less simplification is achieved.
(2) Knowing how many points are left using a threshold before computing is impossible; in other words, setting a proper threshold to maintain the feature of a spectral curve using the necessary points is a difficult and complex task.
(3) For the effective simplification of multiple curves, particularly for hyperspectral images, different thresholds are needed for various curves; moreover, to retain enough points, every curve should set different thresholds.

### 3.3. IDP Algorithm 

To address the problems mentioned above, this paper proposes an improved version of the DP algorithm, which we call IDP. This algorithm does not require setting the threshold but is able to preserve the curve pattern. The major steps are listed below:

(1) Connect the starting point *S* and ending point *E*, calculate the distance from each point to segment *SE*, and retain the point *M*, which has the maximum distance; this is the same as step one in the traditional DP algorithm.
(2) Connect *SM* and *ME*, calculate the distance from each point to segment *SM* and *ME*, and retain the point *N*, which has the maximum distance.
(3) Divide the curve into three parts using points *M* and *N*. Repeat step (2) until it meets the point number retaining requirement.
(4) If the distances are equal in step (2), calculate the ratio of the distance to the line sector and the length of sector, the point with the lower ratio is then retained.

**Figure 4:**
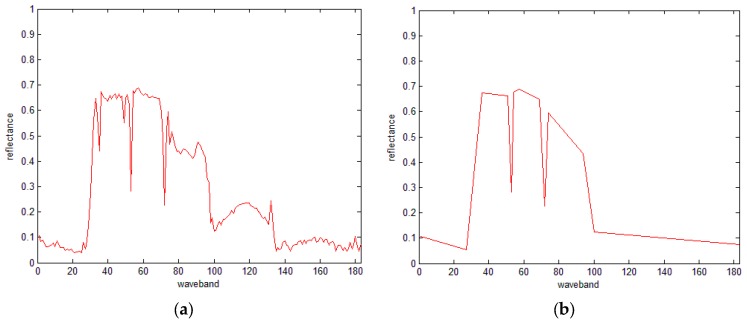

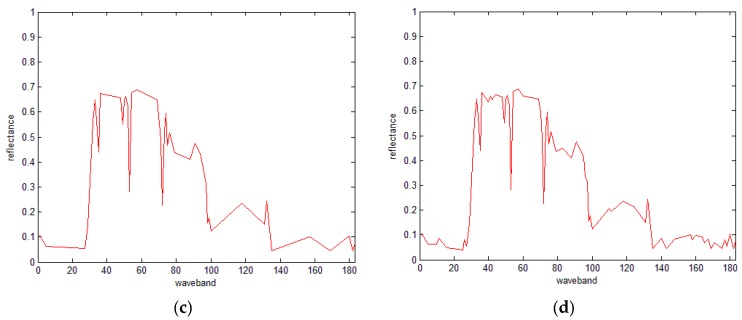
The simplified spectral curves using the IDP algorithm with different numbers. (**a**) The original spectral curve with 183 points, (**b**) 13 retained points, (**c**) 40 retained points, and (**d**) 70 retained points.

In other words, in every iteration, only one point with maximum distance is retained, and once the total number of retained points reach the specified number of points, the iteration stops. Hence, the problems mentioned in the above section could be solved without the threshold. Figure 4 shows the simplified results using different numbers of points. As shown in the figure, the proposed IDP algorithm can well maintain the feature pattern of the spectral curve. Comparing Figure 4b and Figure 3d, we find that the retained points of IDP are the same as those of the traditional DP algorithm, thus indicating the robust results of the proposed IDP.

By specifying the number of retained points, the algorithm can lead to convergence and ensure effective simplification. At the same time, as the threshold is not required, the algorithm can adapt to different curve patterns. 

### 3.4. The IDP Algorithm under SRAI-Restriction

As described in Section 3.1, the SRAI points are essential for representing the basic features of the spectral curve; hence they must be preserved. The IDP algorithm can retain the specified number of points. Combining these two requirements, we propose the IDP algorithm under SRAI-restraint, which consists of the following steps:

(1) Specify the number of SRAI points and then calculate the SRAI feature points.
(2) Specify the number of all the retained points and then set the SRAI points as the initial points of the IDP algorithm. Finally, run the IDP algorithm to determine the remaining points.

**Figure 5:**
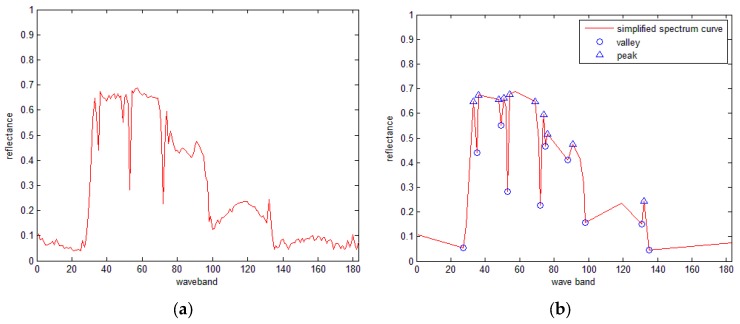

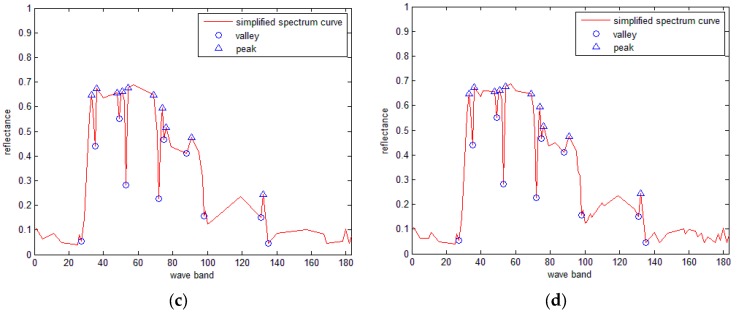
The results of the IDP algorithm under SRAI-restriction with10 peak and 10 valley points. (**a**) The original spectral curve, (**b**) 30 retained points, (**c**) 50 retained points, and (**d**) 70 retained points.

Figure 5 presents the 20 SRAI feature points extracted from the spectral curve. Using the improved algorithm, retention of 30, 50 and 70 feature points are achieved, as shown in Figure 5b–d, respectively, so compared with Figure 5a, the improved algorithm can better preserve the overall pattern of the curve.

### 3.5. SRAI for the Hyperspectral Curves

For hyperspectral curve similarity assessment, the spectral curves are first simplified by SRAI-restrained IDP algorithm, after which the similarity of the simplified curves are compared with the same number of feature points. The calculation of similarity assessment index consists of two steps: band matching and distance calculation:

(1) For the simplified spectral curves *A* and *B*, for a point *A_i_* on curve *A*, if a point *B_j_* on curve B has the same band number as *A_i_*, then *B_j_* is matched with *A_i_*. Repeat this procedure until all points on curve A have been processed. Let variable *N* denote the total number of matched points.
(2) For every matched point, calculate the ED between the reflectance of every matched point, and obtain the sum of all the distances as the final distance.

The final similarity assessment index is thus defined as:

(12) $$Sim={\left(\frac{C}{N}\right)}^{2}\times \sqrt{\frac{1}{N}\sum_{i}^{N}{\left(R_{A\_i}-R_{B\_i}\right)}^{2}}$$

where *N* is the number of matching points and *C* is the number of all feature points; $R_{A\_i}$ and $R_{B\_i}$ are the reflectance of the matched points *A_i_* and *B_i_*, respectively; and $\frac{C}{N}$ is the punishment coefficient. This means that fewer matched points result in higher $\frac{C}{N}$, thus leading to lower similarity. Hence, the lower value of *Sim* indicates the higher similarity of two curves. 

## 4. Experiment and Analysis

To test the performance of the proposed similarity assessment index, two datasets, *i.e.*, AVIRIS dataset and ROSIS dataset were used, and the typical indices, *i.e.*, correlation coefficient (CC), ED, first-order approximation of Kullback-Leibler divergence (KL), SID and SAM, were compared. The mathematical expression of KL is given by:

(13) $$KL=\sum_{i=1}^{N}\frac{{\left[r1(i)-r2(i)\right]}^{2}}{r1(i)+r2(i)}$$

where *r1* and *r2* are the two spectrum curves, respectively.

### 4.1. Airborne Visible Infrared Imaging Spectrometer (AVIRIS) Dataset

**Figure 6:**
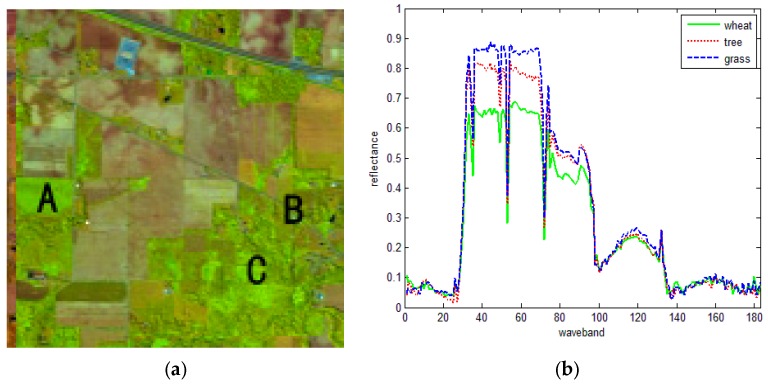
RGB color composite AVIRIS image. (**a**) True color image and (**b**) mean spectral curve of three types of land cover.

The AVIRIS hyperspectral image from the Purdue University Remote Sensing Image Processing Lab was used for our experiments. The original image was captured in June 1992 in an experiment field located in Indiana, USA. The image had 224 bands, the spectral range was from 0.4 to 2.45 μm, the spatial resolution was 20 m, and the image size was 145 × 145 pixels. A total of 183 bands were used for the experiment after removing water absorption and noise bands. Figure 6a presents the RGB composite image of bands 97, 37, and 7.

According to the ground truth, three cover types [*i.e.*, grass (A), wheat (B), and forest (C)] were chosen for the similarity assessment. For simplicity, three pixels of every type were selected for the experiment. Figure 6b shows the similar mean spectral curves of the three types. The evaluation results of our method were compared with those obtained using the CC, ED, SID, KL and SAM methods. The results are listed in Table 1, Table 2, Table 3, Table 4, Table 5 and Table 6, where A1, A2 and A3 correspond to grass; B1, B2 and B3 correspond to wheat; and C1, C2 and C3 correspond to forest. A total of 20 SRAI feature points were exacted and then used to retain 50 points on the spectral curve. The bold back values are the best rank results. In addition, the rank of similarity are listed in the last column. For example, looking at the horizontal view of A3, the similarity between A3 and B2 (0.9977) is bigger than that between A3 and A2 (0.9960), which is obviously wrong (Table 1). Meanwhile, for the rank view of A3, the rank is “A3 > A1 > B2,” where B2 should not be in the third order, thus indicating an error.

**Table 1:** The performance of CC for the AVIRIS dataset.

CC										
	A1	A2	A3	B1	B2	B3	C1	C2	C3	Rank
A1	**1**	**0.9980**	**0.9982**	0.9979	0.9977	0.9967	0.9964	0.9969	0.9968	A1, A3, A2
A2	**0.9980**	**1**	0.9960	**0.9983**	0.9972	0.9962	0.9969	0.9976	0.9968	A2, B1, A1
A3	**0.9982**	0.9960	**1**	0.9967	**0.9977**	0.9973	0.9963	0.9968	0.9968	A3, A1, B2
B1	0.9979	**0.9983**	0.9967	**1**	**0.9983**	0.9968	0.9982	**0.9983**	0.9982	B1, B2, C1/C2
B2	0.9977	0.9972	0.9977	**0.9983**	**1**	**0.9982**	0.9971	0.9978	0.9978	B2, B1, B3
B3	0.9967	0.9962	**0.9973**	0.9968	**0.9982**	**1**	0.9962	0.9969	0.9965	B3, B2, A3
C1	0.9964	0.9969	0.9963	0.9982	0.9971	0.9962	**1**	**0.9994**	**0.9993**	C1, C2, C3
C2	0.9969	0.9976	0.9968	0.9983	0.9978	0.9969	**0.9994**	**1**	**0.9993**	C2, C1, C3
C3	0.9968	0.9968	0.9968	0.9982	0.9978	0.9965	**0.9993**	**0.9993**	**1**	C3, C1, C2

**Table 2:** The performance of ED for the AVIRIS dataset.

ED										
	A1	A2	A3	B1	B2	B3	C1	C2	C3	Rank
A1	**0**	**0.3739**	**0.3280**	0.9241	0.8188	1.0386	1.3415	1.3367	1.2500	A1,A3,A2
A2	0.3739	**0**	**0.3088**	0.6276	**0.3471**	0.8056	1.0458	1.0417	0.9627	A2,A3,B2
A3	**0.3280**	**0.3088**	**0**	0.6942	0.5721	0.7933	1.0870	1.0815	0.9946	A3,A2,A1
B1	0.9241	0.6276	0.6942	**0**	**0.2499**	0.4251	0.4784	0.4823	**0.4068**	B1,B2,C3
B2	0.8188	**0.3471**	0.5721	**0.2499**	**0**	0.4570	0.6084	0.5983	0.4155	B2,B1,A2
B3	1.0386	0.8056	0.7933	**0.4251**	0.4570	**0**	**0.4460**	0.5134	0.4949	B3,B1,C1
C1	1.3415	1.0458	1.0870	0.4784	0.6084	0.4460	**0**	**0.1618**	**0.1800**	C1,C2,C3
C2	1.3367	1.0417	1.0815	0.4823	0.5983	0.5134	**0.1618**	**0**	**0.1874**	C2,C1,C3
C3	1.2500	0.9627	0.9946	0.4068	0.4155	0.4949	**0.1800**	**0.1874**	**0**	C3,C1,C2

**Table 3:** The performance of SID for the AVIRIS dataset.

SID										
	A1	A2	A3	B1	B2	B3	C1	C2	C3	Rank
A1	**0**	**0.0087**	**0.0066**	0.0154	0.0183	0.0101	0.0159	0.0109	0.0147	A1,A3,A2
A2	0.0087	**0**	**0.0082**	0.0085	0.0114	0.0141	0.0105	**0.0064**	0.0117	A2,A3,C2
A3	**0.0066**	**0.0082**	**0**	0.0110	0.0126	0.0117	0.0112	0.0083	0.0109	A3,A1,A2
B1	0.0154	0.0085	0.0110	**0**	0.0078	0.0210	**0.0059**	**0.0069**	0.0076	B1,C1,C2
B2	0.0183	0.0114	0.0126	**0.0078**	**0**	0.0214	0.0100	0.0085	**0.0075**	B2,C3,B1
B3	**0.0101**	0.0141	**0.0117**	0.0210	0.0214	**0**	0.0219	0.0150	0.0214	B3,A1,A3
C1	0.0159	0.0105	0.0112	**0.0059**	0.0100	0.0219	**0**	**0.0071**	0.0078	C1,B1,C2
C2	0.0109	**0.0064**	0.0083	**0.0069**	0.0085	0.0150	0.0071	**0**	0.0076	C2,A2,B1
C3	0.0147	0.0117	0.0109	**0.0076**	**0.0075**	0.0214	0.0078	**0.0076**	**0**	C3,B2,B1/C2

**Table 4:** The performance of KL for the AVIRIS dataset.

KL										
	A1	A2	A3	B1	B2	B3	C1	C2	C3	Rank
A1	**0**	**0.2441**	**0.2080**	0.8186	0.7634	1.3321	1.4262	1.4227	1.2981	A1,A3,A2
A2	0.2441	**0**	**0.2109**	0.4512	**0.2323**	1.1203	0.9707	0.9656	0.9177	A2,A3,B2
A3	**0.2080**	**0.2109**	**0**	0.4835	0.4373	0.9866	0.9297	0.9518	0.8453	A3,A1,A2
B1	0.8186	0.4512	0.4835	**0**	**0.2207**	0.7410	**0.2934**	0.3687	0.3044	B1,B2,C1
B2	0.7634	**0.2323**	0.4373	**0.2207**	**0**	0.8417	0.4877	0.5064	0.3786	B2,B1,A2
B3	1.3321	1.1203	0.9866	0.7410	0.8417	**0**	0.6529	**0.4534**	**0.6356**	B3,C1,C2
C1	1.4262	0.9707	0.9297	0.2934	0.4877	0.6529	**0**	**0.1272**	**0.1388**	C1,C2,C3
C2	1.4227	0.9656	0.9518	0.3687	0.5064	0.4534	**0.1272**	**0**	**0.1473**	C2,C1,C3
C3	1.2981	0.9177	0.8453	0.3044	0.3786	0.6356	**0.1388**	**0.1473**	**0**	C3,C1,C2

**Table 5:** The performance of SAM for the AVIRIS dataset.

SAM										
	A1	A2	A3	B1	B2	B3	C1	C2	C3	Rank
A1	**1**	**0.9988**	**0.9992**	0.9981	0.9980	0.9986	0.9972	0.9979	0.9976	A1,A3,A2
A2	0.9988	**1**	0.9982	**0.9991**	0.9986	0.9977	0.9983	**0.9989**	0.9984	A2,B1,C2
A3	**0.9992**	0.9982	**1**	0.9980	0.9985	**0.9986**	0.9977	0.9982	0.9981	A3,A1,B3
B1	0.9981	0.9991	0.9980	**1**	0.9991	0.9975	**0.9992**	**0.9993**	**0.9992**	B1,C2,C1/C3
B2	0.9980	0.9986	0.9985	**0.9991**	**1**	0.9977	0.9987	**0.9990**	**0.9990**	B2,B1,C2/C3
B3	**0.9986**	0.9977	**0.9986**	0.9975	0.9977	**1**	0.9966	0.9975	0.9970	B3,A1,A3
C1	0.9972	0.9983	0.9977	**0.9992**	0.9987	0.9966	**1**	**0.9990**	0.9987	C1,B1,C2
C2	0.9979	0.9989	0.9982	**0.9993**	0.9990	0.9975	0.9990	**1**	**0.9991**	C2,B1,C3
C3	0.9976	0.9984	0.9981	**0.9992**	0.9990	0.9970	0.9987	**0.9991**	**1**	C3,B1,C2

**Table 6:** The performance of the proposed index for the AVIRIS dataset.

Proposed										
	A1	A2	A3	B1	B2	B3	C1	C2	C3	Rank
A1	**0**	**0.0637**	**0.0748**	0.1495	0.1960	0.2151	0.3167	0.2271	0.2295	A1,A2,A3
A2	**0.0637**	**0**	**0.0568**	0.1202	0.1343	0.1516	0.1829	0.1678	0.1838	A2,A3,A1
A3	**0.0748**	**0.0568**	**0**	0.1797	0.1412	0.2417	0.1819	0.2116	0.2304	A3,A2,A1
B1	0.1495	0.1202	0.1797	**0**	**0.0518**	**0.0726**	0.1101	0.1001	0.0934	B1,B2,B3
B2	0.1960	0.1343	0.1412	**0.0518**	**0**	**0.0883**	0.1509	0.1445	0.1443	B2,B1,B3
B3	0.2151	0.1516	0.2417	**0.0726**	**0.0883**	**0**	0.1122	0.1242	0.0966	B3,B1,B2
C1	0.3167	0.1829	0.1819	0.1101	0.1509	0.1122	**0**	**0.0293**	**0.0412**	C1,C2,C3
C2	0.2271	0.1678	0.2116	0.1001	0.1445	0.1242	**0.0293**	**0**	**0.0461**	C2,C1,C3
C3	0.2295	0.1838	0.2304	0.0934	0.1443	0.0966	**0.0412**	**0.0461**	**0**	C3,C1,C2

From Figure 6b, we can see that these three types can be easily confused with one another. For SID results, there are 11 confusing pairs (*i.e.*, A2 and C2, B1 and C1, B1 and C2, B2 and C3, B3 and A1, B3 and A3, C1 and B1, C2 and A2, C2 and B1, C3 and B2, C3 and B1), which can also be seen from the rank column. For the SAM results, there are 13 confusing pairs (*i.e.*, A2 and B1, A2 and C2, A3 and B3, B1 and C2, B1 and C1/C3, B2 and C2/C3, B3 and A1, B3 and A3, C1 and B1, C2 and B1, C3 and B1). For the CC, ED and KL indices, there are five, four and five confusing pairs, respectively. In comparison, for the proposed index, all the types are clearly distinguished, which is also reflected in both the similarity assessment values and ranking. The results indicate that the proposed index is effective in distinguishing different vegetation types with similar spectra.

### 4.2. Reflective Optics System Imaging Spectrometer (ROSIS) Dataset

**Figure 7:**
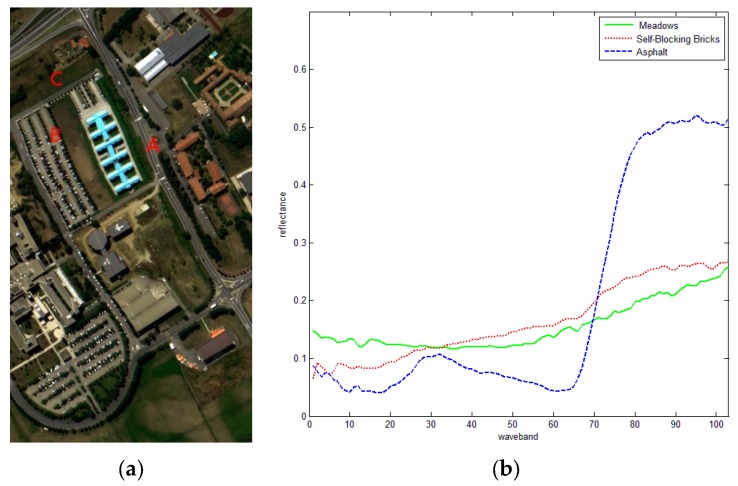
RGB color composite ROSIS image. (**a**) True color image and (**b**) mean spectral curve of three types of land cover.

The ROSIS dataset was acquired using Reflective Optics System Imaging Spectrometer-DLR (ROSIS_03), Schneider Systemtechnik-HySens Pavia Campaign (2002). The data set acquired from the ROSIS sensor does not fully cover the areas of interest because of the narrower field of view of the DAIS instrument on which the flight lines have been designed. Data were atmospherically corrected but not geometrically corrected. The number of spectral bands was 103 for the “University” dataset, and geometric resolution was 1.3 meters. The images were acquired during a flight campaign over Pavia, in northern Italy (45°11’ N, 9°9’ E), on the 8th of July 2002 from 10:30 a.m. to 12:00 noon. Figure 7a presents the RGB composite image.

According to the ground truth land cover map, three cover types, *i.e.*, Asphalt (A), Self-Blocking Bricks (B), and Meadows (C), on the image were chosen for the similarity assessment. For simplicity, three pixels of every type were selected for the experiment. Figure 7b shows the mean spectral curves of the three types, from which it can be observed that Asphalt and Self-Blocking Bricks are similar while Meadows is different. The results of SID, SAM, and the proposed index are listed in Table 4, Table 5 and Table 6, respectively, in which A1, A2 and A3 denote Asphalt; B1, B2 and B3 denote Self-Blocking Bricks; and C1, C2 and C3 denote Meadows. A total of 50 points were retained on the spectral curve, and 10 SRAI feature points were selected. The bold back values show the best ranking results. In addition, the similarity rankings are listed in the last column.

Similar to Figure 6b, in Figure 7b, the spectral curves of cover types A and B can be easily confused with each other. In Table 7, Table 8 and Table 9 there are nine confusing pairs in total. Table 10 shows three confusing pairs (A2 and B2, A3 and B1, B2 and A2), while Table 11 shows five confusing pairs (A2 and B3, A3 and B2, B1 and A2, B2 and A3, B3 and A2). These results are also shown in the ranking columns. In comparison, in Table 12, all cover types are clearly distinguished, indicating that the proposed index shows better performance compared with CC, ED, KL, SID and SAM.

**Table 7:** The performance of CC for the ROSIS dataset.

CC										
	A1	A2	A3	B1	B2	B3	C1	C2	C3	Rank
A1	**1.0000 **	**0.9775 **	**0.9798 **	0.9024	0.9156	0.9054	0.9441	0.9376	0.9437	A1,A3,A2
A2	**0.9775 **	**1.0000 **	0.9645	0.9570	**0.9673 **	0.9579	0.9362	0.9327	0.9382	A2,A1,B2
A3	**0.9798 **	**0.9645 **	**1.0000 **	0.9560	0.9643	0.9593	0.9392	0.9350	0.9406	A3,A1,A2
B1	0.9024	**0.9570 **	0.9560	**1.0000 **	0.9524	**0.9961 **	0.9128	0.9152	0.9176	B1,B3,A2
B2	0.9156	**0.9673 **	0.9643	0.9524	**1.0000 **	**0.9937 **	0.9243	0.9264	0.9278	B2,B3,A2
B3	0.9054	0.9579	0.9593	**0.9961 **	**0.9937 **	**1.0000 **	0.9202	0.9223	0.9245	B3,B1,B2
C1	0.9441	0.9362	0.9392	0.9128	0.9243	0.9202	**1.0000 **	**0.9991 **	**0.9994 **	C1,C3,C2
C2	0.9376	0.9327	0.9350	0.9152	0.9264	0.9223	**0.9991 **	**1.0000 **	**0.9994 **	C2,C3,C1
C3	0.9437	0.9382	0.9406	0.9176	0.9278	0.9245	**0.9994 **	**0.9994 **	**1.0000 **	C3,C1,C2

**Table 8:** The performance of ED for the ROSIS dataset.

ED										
	A1	A2	A3	B1	B2	B3	C1	C2	C3	Rank
A1	**0.0000 **	**0.1923**	0.4887	**0.3429**	0.4139	0.4501	1.5770	2.0089	1.6241	A1,A2,B1
A2	**0.1923**	**0.0000 **	**0.0787**	0.2705	0.3597	0.3867	1.4922	1.9360	1.5395	A2,A3,A1
A3	0.4887	**0.0787**	**0.0000 **	**0.3112**	0.4091	0.4314	1.5348	1.9820	1.5831	A3,A2,B1
B1	0.3429	**0.2705**	0.3112	**0.0000 **	0.3444	**0.1491**	1.3785	1.7978	1.4199	B1,B3,A2
B2	0.4139	0.3597	0.4091	**0.3444**	**0.0000 **	**0.0904**	1.3043	1.7101	1.3443	B2,B3,B1
B3	0.4501	0.3867	0.4314	**0.1491**	**0.0904**	**0.0000 **	1.2787	1.6847	1.3175	B3,B2,B1
C1	1.5770	1.4922	1.5348	1.3785	1.3043	1.2787	**0.0000 **	**0.4871**	**0.0842**	C1,C3,C2
C2	2.0089	1.9360	1.9820	1.7978	1.7101	1.6847	**0.4871**	**0.0000 **	**0.4313**	C2,C3,C1
C3	1.6241	1.5395	1.5831	1.4199	1.3443	1.3175	**0.0842**	**0.4313**	**0.0000 **	C3,C1,C2

**Table 9:** The performance of SID for the ROSIS dataset.

SID										
	A1	A2	A3	B1	B2	B3	C1	C2	C3	Rank
A1	**0.0000 **	**0.0165 **	**0.0190 **	0.0468	0.0456	0.0594	0.5079	0.5212	0.5029	A1,A2,A3
A2	0.0165	**0.0000 **	**0.0020 **	0.0166	**0.0138 **	0.0224	0.3945	0.4035	0.3859	A2,A3,B2
A3	0.0190	**0.0020 **	**0.0000 **	**0.0018 **	0.0164	0.0234	0.4069	0.4172	0.3992	A3,A2,B1
B1	0.0468	0.0166	**0.0018 **	**0.0000 **	0.0040	**0.0028 **	0.3804	0.3839	0.3682	B1,B3,B2
B2	0.0456	0.0138	0.0164	**0.0040 **	**0.0000 **	**0.0046 **	0.3675	0.3703	0.3568	B2,A2,B1
B3	0.0594	0.0224	0.0234	**0.0028 **	**0.0046 **	**0.0000 **	0.3516	0.3542	0.3395	B3,B2,B1
C1	0.5079	0.3945	0.4069	0.3804	0.3675	0.3516	**0.0000 **	**0.0035 **	**0.0028 **	C1,C3,C2
C2	0.5212	0.4035	0.4172	0.3839	0.3703	0.3542	**0.0035 **	**0.0000 **	**0.0029 **	C2,C3,C1
C3	0.5029	0.3859	0.3992	0.3682	0.3568	0.3395	**0.0028 **	**0.0029 **	**0.0000 **	C3,C1,C2

**Table 10:** The performance of KL for the ROSIS dataset.

KL										
	A1	A2	A3	B1	B2	B3	C1	C2	C3	Rank
A1	**0.0000**	**0.1542**	**0.1540**	0.4020	0.4914	0.5996	4.7123	6.0548	4.8043	A1,A3,A2
A2	0.1542	**0.0000**	**0.0203**	0.3606	**0.0828**	0.4234	3.9258	5.3117	3.9991	A2,A3,B1
A3	0.1540	**0.0203**	**0.0000**	**0.0955**	0.4606	0.5081	4.1037	5.5636	4.1915	A3,A2,A1
B1	0.4020	0.3606	0.0955	**0.0000**	**0.0916**	**0.0542**	3.5761	4.6501	3.5883	B1,B3,A2
B2	0.4914	**0.0828**	0.4606	0.0916	**0.0000**	**0.0411**	3.4130	4.3212	3.4172	B2,B3,B1
B3	0.5996	0.4234	0.5081	**0.0542**	**0.0411**	**0.0000**	3.2701	4.1749	3.2621	B3,B2,B1
C1	4.7123	3.9258	4.1037	3.5761	3.4130	3.2701	**0.0000**	**0.3048**	**0.0321**	C1,C3,C2
C2	6.0548	5.3117	5.5636	4.6501	4.3212	4.1749	**0.3048**	**0.0000**	**0.2392**	C2,C3,C1
C3	4.8043	3.9991	4.1915	3.5883	3.4172	3.2621	**0.0321**	**0.2392**	**0.0000**	C3,C1,C2

**Table 11:** The performance of SAM for the ROSIS dataset.

SAM										
	A1	A2	A3	B1	B2	B3	C1	C2	C3	Rank
A1	**1.0000**	**0.9932**	**0.9947**	0.9841	0.9845	0.9805	0.8631	0.8601	0.8628	A1,A3,A2
A2	0.9932	**1.0000**	**0.9993**	0.9950	0.9954	**0.9991**	0.9009	0.8985	0.9011	A2,A3,B3
A3	0.9947	**0.9993**	**1.0000**	0.9940	**0.9989**	0.9930	0.8967	0.8941	0.8968	A3,A2,B2
B1	0.9841	0.9950	0.9940	**1.0000**	**0.9948**	0.9933	0.9014	0.9006	0.9025	B1,A2,B2
B2	0.9845	0.9954	**0.9989**	0.9948	**1.0000**	**0.9989**	0.9063	0.9054	0.9070	B2,B3,A3
B3	0.9805	**0.9991**	0.9930	0.9933	**0.9989**	**1.0000**	0.9114	0.9106	0.9124	B3,A2,B2
C1	0.8631	0.9009	0.8967	0.9014	0.9063	0.9114	**1.0000**	**0.9996**	**0.9997**	C1,C3,C2
C2	0.8601	0.8985	0.8941	0.9006	0.9054	0.9106	**0.9996**	**1.0000**	**0.9997**	C2,C3,C1
C3	0.8628	0.9011	0.8968	0.9025	0.9070	0.9124	**0.9997**	**0.9997**	**1.0000**	C3,C1,C2

**Table 12:** The performance of the proposed index for the ROSIS dataset.

Proposed										
	A1	A2	A3	B1	B2	B3	C1	C2	C3	Rank
A1	**0.0000**	**0.0829**	**0.0622**	0.1473	0.1192	0.2132	0.5461	0.9955	0.6399	A1,A3,A2
A2	**0.0829**	**0.0000**	**0.0339**	0.1180	0.1333	0.1645	0.4875	1.0929	0.5742	A2,A3,A1
A3	**0.0622**	**0.0339**	**0.0000**	0.1149	0.1514	0.1349	0.6716	0.6859	0.4885	A3,A2,A1
B1	0.1473	0.1180	0.1149	**0.0000**	**0.0510**	**0.0736**	0.6502	0.7601	0.4397	B1,B2,B3
B2	0.1192	0.1333	0.1514	**0.0510**	**0.0000**	**0.0501**	0.5167	0.5091	0.5821	B2,B3,B1
B3	0.2132	0.1645	0.1349	**0.0736**	**0.0501**	**0.0000**	0.4990	0.5677	0.3716	B3,B2,B1
C1	0.5461	0.4875	0.6716	0.6502	0.5167	0.4990	**0.0000**	**0.1852**	**0.0376**	C1,C3,C2
C2	0.9955	1.0929	0.6859	0.7601	0.5091	0.5677	**0.1852**	**0.0000**	**0.1232**	C2,C3,C1
C3	0.6399	0.5742	0.4885	0.4397	0.5821	0.3716	**0.0376**	**0.1232**	**0.0000**	C3,C1,C2

### 4.3.The Impacts of the Parameters

**Figure 8:**
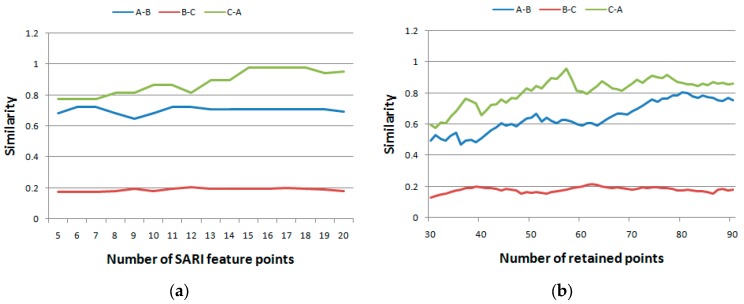
The impacts of parameters on the AVIRIS dataset. (**a**) Impact on the number of SRAI feature points and (**b**) on the number of retained points.

**Figure 9:**
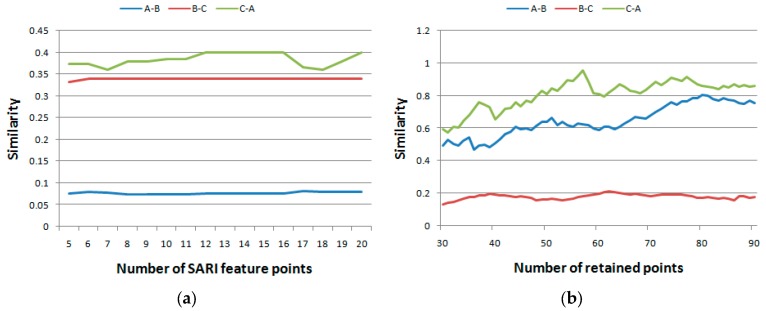
The impacts of parameters on the ROSIS dataset. (**a**) Impact on the number of SRAI feature points and (**b**) on the number of retained points.

There are two parameters, *i.e.*, the number of retained points and the number of SRAI feature points, in the previous experiments. In this experiment, the impacts of these two parameters were tested. The mean spectral curves of the AVIRIS dataset used in the first experiment (A, B, and C) were used, after which the similarity index using different numbers of retained points and number of SRAI feature points were calculated. When testing the impact of the number of retained points, the number of SRAI feature points was kept at 10, and the number of retained points increased from 30 to 90. In every increasing step, the similarities of A and B, B and C, and C and A were calculated. Meanwhile, when testing the impact of number of SRAI feature points, the number of retained points was kept at 50, whereas the number of SRAI feature points increased from 5 to 20. The above three similarities were calculated. The results are shown in Figure 8 and Figure 9, respectively. As shown in the figures, the x axis indicates the number of retained points or SRAI feature points, and the y axis shows the similarities among A, B, and C.

Analogous to the AVIRIS dataset in Figure 8, the ROSIS dataset was also used to demonstrate the impacts of the two parameters of the proposed method. The results are shown in Figure 9.

From Figure 8 and Figure 9, we can see that a higher number of retained points does not necessarily imply a higher number of matched points; hence, the value of the similarity index may be different. No matter how the similarity index values changed, the similarity trends were coincident, as shown in Figure 8 and Figure 9. These results indicated that for practical use, any number of SRAI feature points or retained points could be set. However, as a more proper choice, we can choose a point at which the difference between similarities is the largest. 

## 5. Conclusions

Spectral similarity assessment is a basic issue in hyperspectral image classification and object recognition. As the most intuitive way to express the spectral features, the spectrum curve and its features have always been investigated in the literature. Given that a hyperspectral image usually contains hundreds of bands, reducing the dimensions of a hyperspectral image is an important research topic. 

As a solution, the DP algorithm can be used in a single spectrum curve and, therefore, is more suitable for single spectrum curve similarity comparison. However, given that there is a distance threshold in the traditional DP algorithm, it is difficult to determine the most proper threshold, which can retain enough points to complete the similarity assessment. 

This paper proposes an improved DP algorithm to solve this problem. After setting the number of retained points, the proposed method could automatically extract the expected points without using the threshold. This algorithm can consistently achieve convergence, resulting in a higher number of extracted points that, in turn, facilitates a more effective similarity assessment. As the reflection and absorption features are important for spectral recognition, SRAI is introduced to restrain the simplification process. Finally, enough points (including the important SRAI features) are obtained for the proposed method. 

To test the performance of the proposed method, two widely used hyperspectral image datasets, *i.e.*, AVIRIS and ROSIS, were used. The assessment results were compared with the results of existing indices, *i.e.*, CC, ED, SID, SAM, and KL. The experiment results indicate that the existing indices may lead to obvious incorrect results, especially when the testing curves are similar. In comparison, our proposed method could achieve high accuracy using fewer points, because the most import SRAI features are retained. Finally, the impacts of the number of SRAI points and the number of all retained points have been analyzed. Further research may focus on investigating the practicality of this method.
